# Clinical examination factors that predict delayed recovery in individuals with concussion

**DOI:** 10.1186/s40945-020-00081-z

**Published:** 2020-05-26

**Authors:** Corina Martinez, Zachary Christopherson, Ashley Lake, Heather Myers, Jeffrey R. Bytomski, Robert J. Butler, Chad E. Cook

**Affiliations:** 1 Department of Physical Therapy and Occupational Therapy, Duke University, DUMC 3965, Durham, NC 27705 USA; 2 Department of Orthopedics, Duke University, Durham, NC USA; 3 St Louis Cardinals, St. Louis, MO USA; 4 Division of Physical Therapy, Department of Orthopedics, Duke University, 2200 W. Main Street, Durham, NC 27708 USA

**Keywords:** Concussion, Post-concussion syndrome, Vestibular

## Abstract

**Background:**

Risk factors for prolonged recovery after concussion have been well researched, but specific objective clinical examination findings have not. This study examined whether clinical examination results could predict delayed recovery (DR) in individuals with concussion diagnosis. A secondary aim explored the influence of early examination on individual prognosis.

**Methods:**

The study was a retrospective, observational cohort design that included 163 individuals seen at a concussion clinic who were followed longitudinally until cleared for sports activity. Cognitive, visual, balance, vestibular, and cervical clinical testing and symptom assessment were performed at initial evaluation. DR was calculated by taking the median value associated with time to clearance for activity. Bivariate logistic regression analysis was calculated to determine odds ratios (and 95% confidence intervals) for the odds of DR with presence or absence of each clinical finding. Multivariate analyses were used to define the best predictors of DR.

**Results:**

80 of 163 individuals were considered delayed in their clearance to activity. Cognitive impairments (OR = 2.72; 95%CI = 1.40, 5.28), visual exam findings (OR = 2.98; 95%CI = 1.31, 6.80), and vestibular exam findings (OR = 4.28; 95%CI = 2.18, 8.43) all increased the odds of a DR. Multivariate modeling retained cognitive symptoms and clinical examination-vestibular testing as predictors of delayed recovery. Time to examination after injury was a mediator for DR.

**Conclusions:**

The clinical examination provides value in identifying individuals who are likely to exhibit a delayed clearance. In particular, vestibular impairments identified clinically at initial evaluation and cognitive symptoms were associated with increased odds of a DR to return to activity. Our data support that early implementation of a standardized clinical examination can help to identify individuals who may be more at risk of prolonged recovery from concussion.

## Background

The incidence of sports-related concussion (SRC) in high school and collegiate athletes is reported to be 0.11 and 0.28, respectively, per 1000 athlete-exposures, a number that has steadily risen over the last decade [1]. This accounts for an estimated 1.6 to 3.8 million concussions annually [2] and equates to approximately 5% of the total number of injuries across different sports with American football, girls’ soccer, boys’ soccer, and girls’ basketball resulting in the majority of concussion in high school athletes [1]. Of these nearly 4 million concussions, around 300,000 involve youth athletes aged 14–19 [3], with visits to the emergency department for pediatric sports-related traumatic brain injuries increasing 60% over the previous 10 years [4].

The majority of individuals who suffer from sports-related concussion fully recover within a few days to several weeks [5]. However, there is a portion of the population who experience symptoms beyond the generally accepted time frame for recovery. Recovery is interpreted as resolution of symptoms and clearance to return to sport activity. This condition is commonly referred to as post-concussion syndrome (PCS), a term used to describe the presence of nonspecific signs and symptoms that are linked to several possible causes that do not necessarily reflect ongoing physiological brain injury [6]. There is a paucity in the literature regarding the time frame for PCS. Williams et al. found that high school athletes tend to report longer symptom recovery than college athletes [3]. A recent 4-year study following NCAA athletes found that most symptoms associated with concussion resolved within 1 week (60.1%); however, 6.2% had a resolution of symptoms of over 4 weeks, and 8.9% of concussions required over 4 weeks before return to play [2].

A multitude of risk factors for developing PCS have been identified in the literature including severity of initial symptoms, prolonged headache, subjective concentration deficits, female gender, referral to a concussion rehabilitation program, history of prior concussion, dizziness, vestibulo-ocular dysfunction and younger age [7–15]. Others have also reported history of psychological (e.g. depression, anxiety) or neurodevelopmental (e.g. attention-deficit/hyperactivity disorder [ADHD], learning disability [LD]) disorders as risk factors for developing PCS [2, 4, 16–18]. Most of the research related to determining which athletes will have a resolution of symptoms has been focused on correlations to performance on computerized neuro-cognitive/neuropsychological testing and formal sideline testing [5, 17–20], with little attention paid to the relationship between PCS and clinical examination findings.

The clinical examination used to assess concussion is variable based on the preferences and experiences of the clinician because there are no established guidelines on required components. In general, it will consist of the following: a detailed history, neurological examination, cognitive examination including neurocognitive screening, cervical spine screening, balance testing, vestibular-ocular motor screening, and some form of reaction time testing [21, 22]. Prognostic information regarding risk factors is important because it allows clinicians to be more confident in management decisions regarding return to play and gives perspective regarding the potential for delayed recovery. However, little is known about the correlation between clinical examination findings and prolonged recovery.

The timing of former clinical examination following a concussive injury is another factor that has not been well researched. Diagnosis of concussion is typically made acutely by licensed athletic trainers, team physicians, emergency department providers, or urgent care providers using commonly accepted sideline concussion diagnostic tools such as the Sideline Concussion Assessment Tool or the King-Devick Test [23, 24]. Following the acute concussion diagnosis, referral to a licensed healthcare provider is consistently recommended, however the optimal timing for clinical follow-up is not established.

The aim of this study was to examine whether clinical examination results could predict delayed recovery in individuals with concussion symptoms who were seen acutely at a concussion clinic. A secondary aim was to explore the role of early examination and how this influences the prognosis of the individual.

## Methods

### Reporting guidelines

The study used the *STrengthening the Reporting of OBservational studies in Epidemiology* (STROBE) guidelines for reporting of cohort studies. STROBE is endorsed by a growing number of biomedical journals and aims to improve reporting standards of observational studies [25].

### Study design

The study was a retrospective, observational cohort design that evaluated subjects after exposure to a clinically diagnosed concussion based on mechanism of injury, symptom report, and clinical presentation as defined by the international consensus statement on concussion in sport [23]. Follow-up data were captured at a number of time-points including baseline (post-exposure), follow-up and discharge. The study protocol (ID Pro00058398) was approved by the institutional review board at the medical center where the study was conducted.

### Setting

All data were captured at a sports concussion clinic that operates within the Duke Sports Sciences Institute (DSSI). The DSSI is a multidisciplinary facility with medical and rehabilitation management options for sports related injury and non-injury management. The concussion clinic is staffed by four primary care sports medicine physicians trained in concussion management. All individuals with concussion were evaluated by one of these physicians and clinical decision making was based on established protocols supported by the literature. The evaluation required the individual to complete subjective questionnaires including the Post-Concussion Symptom Scale, The Neck Disability Index, and the Dizziness Handicap Index. The clinical examination was comprised of standardized concussion tests including the Standardized Assessment of Concussion (SAC) for cognitive assessment, King-Devick Test for visual assessment, Vestibular-Ocular Motor Screen (VOMS) for visual (smooth pursuit and convergence) and vestibular (saccades, VOR, VMS) assessment, and the Balance Error Scoring System (BESS) for balance assessment. Cervical testing was considered positive if neck pain was present at rest, with palpation, or with active motion. Standard clinical concussion care was provided to all individuals based on their initial presentation.

### Participants

Participants included 163 adolescent and college aged individuals who self-presented or were referred for evaluation at the sports concussion clinic at the Duke Sports Sciences Institute between July 1, 2013 and January 1, 2015. Eligibility required a medical diagnosis of sports-related concussion and the willingness to receive a battery of questionnaires, examinations, and follow up. Follow up occurred through January 22, 2015 as indicated by standard of care.

### Variables

Descriptive characteristics included age, sex, Neck Disability Index score (NDI), Dizziness Handicap Index score (DHI), Post-Concussion Symptom Scale (PCSS), and time from concussion incident to clinical examination. The NDI is a 10-item questionnaire that identifies the presence of neck pain with daily activities such as personal care, reading, lifting, driving, sleeping, and work and is scored as a percentage disability [26]. The DHI asks the subject to rate self-perceived dizziness or imbalance difficulties during 25 daily activities which may be impacted by vestibular involvement [27]. The Post-Concussion Symptom Scale is a subjective rating of the severity of 22 common post-concussive symptoms scored on a 7 point (Likert) scale with the maximum value being 132. The PCSS is a commonly used self-assessment rating of concussion symptoms that has been shown to be predictive of concussive injuries. The symptoms on the PCSS can be categorized into 4 domains which include physical, cognitive, emotional, and sleep disturbances [28, 29].

Predictor variables included the presence or absence of 1) headache, 2) dizziness, 3) neck pain, 4) cognitive impairments, 5) photophobia, 6) phonophobia, and 7) vision disturbances as a primary symptom. The above variables were determined by subjective history taken at the beginning of the evaluation and were considered positive if individual endorsed presence of the symptom during the acute phase of the concussion. Predictors also included a series of standard clinical examinations for cognitive testing, cervical screening, visual testing, balance testing, and vestibular testing (Table 1). Clinical examination data were calculated as ‘positive’ or ‘negative’. An additional predictor was “time to examination”, which was the difference between the first examination date and the original injury date. Time to examination was dichotomized by median values to improve the understanding of the analyses and to examine the necessity of a sub-classification of time to examination.

The outcome variable for this study was time to clearance. Time to clearance was calculated by subtracting the numbers of days from the date of clearance by the date of examination. The resulting value was in ‘days’. Time to clearance was dichotomized by median values to improve the understanding of the analyses and to create odds ratios for each predictor variable. Those with days above the median were defined as “delayed recovery” whereas those with days below the median were defined as “non-delayed recovery”.

**Table 1:** Clinical examination tests utilized at initial evaluation

System	Assessment Method	Abnormal Findings	Reference
Cognitive	Standardized Assessment of Concussion (SAC)	Score < 26, mean score 26.6 (1996 McCrea)	McCrea M, Kelly J, Randolph C. Standardized Assessment of Concussion (SAC): Manual for Administration, Scoring and Interpretation. Waukesha, WI: CNS Inc.; 1996.
Cervical	Active ROM Palpation of spinous processes and musculature	Pain Decreased ROM	
Visual	King-Devick (KD)	Symptom provocation during test	Galetta KM, Barrett J, Allen M, et al. The King-Devick test as a determinant of head trauma and concussion in boxers and MMA fighters. *Neurology*. 2011;76 (17):1456–1462. doi:10.1212/WNL.0b013e31821184c9.
Balance	Balance Error Scoring System (BESS)	Score > 19, normal scores 12.03 ± 7.34	Guskiewicz KM. Postural stability assessment following concussion: One piece of the puzzle. *Clin J of Sport Med*. 2001;11 (3):182–189.
Vestibular	Vestibular Oculomotor Screen (VOMS): (1) smooth pursuit, (2) horizontal and vertical saccades*, (3) near point of convergence (NPC) distance, (4) horizontal vestibular ocular reflex (VOR)*, and (5) visual motion sensitivity (VMS) *Performed for 10 s	Symptom provocation with any of the tests	Mucha A, Collins MW, Elbin RJ, et al. A Brief Vestibular/Ocular Motor Screening (VOMS) Assessment to Evaluate Concussions: Preliminary Findings*. Am J Sports Med.* 2014;42:2479–2486.

### Bias

To decrease risk of bias the statistician was different than the clinicians and database stewards of the study.

### Study size

For simple univariate multinomial or logistic regression, Homer and Lemeshow have recommended a minimum observation-to-variable ratio of 10:1, but cautioned that a number this low will likely overfit a model [30]. Their preferred observation-to-variable ratio is 20:1 for the multivariate modeling, thus an appropriate number for multivariate modeling would range 120 to 240 if all predictor variables were eligible for the final analysis [30].

### Statistical methods

All analyses were performed using SPSS version 20.0 (IBM Corp. Armonk, NY, USA). Subject characteristics, including means, standard deviations, and frequencies were reported for age, gender, disability, dizziness, symptom statuses and time to examination. Descriptive characteristics were reported in raw values. All patient report data were categorized as present or absent.

Univariate logistic regression analyses were performed for each of the predictor variables using the outcome variable of time to clearance. Nuisance variables for history of concussion, LD/ADHD and migraines were included in each regression. Logistic regression analysis was used because the pass rates were not normally distributed, could not be appropriately log-transformed, and failed to meet the assumptions of a linear regression analysis. For each univariate analysis, individual *P*-values, odds ratios (ORs) and 95% confidence intervals (CIs), were reported.

Associations in the univariate analyses with P-values ≤0.05 were considered in a distinct hierarchical multivariate predictive model which included the full sample. After assessment of collinearity, multivariate analyses (backwards stepwise regression) were used to define the best predictors of delayed recovery. Secondary analyses included multivariate modeling for two subgroups: 1) delayed time to examination and 2) early time to examination, to determine the influence of examination timing on predictive outcomes.

### Availability of data and materials

The datasets analyzed during the current study are not publicly available due to institutional restrictions regarding the accessibility of private health information. A limited dataset, with HIPAA identifiers removed, may be available from the corresponding author on reasonable request.

## Results

### Participants

The sample was mostly younger (mean age = 16.2 SD = 3.63) males (65%), who most frequently were seen after a direct hit to the head (55.3%) and most commonly included American football players (33.1%). The mean time from injury to examination in days was 10.4 (SD = 27.2), range 0 to 256 days (Q1 = 2; med = 4; Q3 = 7.5). The mean time from injury to clearance (in days) was 29.63 (SD = 97.93), range 0–422 days (Q1 = 4; med = 9; Q3 = 20). The study included 163 individuals who were initially seen for their concussion in the 2014 calendar year and tracked until full resolution of their concussion symptoms. Eighty (80) were considered delayed in their clearance to activity taking 29 days or more to recover from their concussion. Those with delayed clearance to activity also had longer days between injury and examination (Table 2).

**Table 2:** Descriptive characteristics of the sample (*N* = 163)

Variables	Total Sample	Individuals with a Delayed Recovery and Clearance	Individuals with a Non-Delayed Recovery and Clearance
Gender	57 = female 106 = male	34 = Female 46 = Male	23 = Female 60 = Male
Age	16.25 (3.63)	16.47 (4.36)	16.02 (2.81)
Neck Disability Index Score (%)	21.65 (17.81)	21.80 (14.30)	21.21 (26.23)
Dizziness Handicap Inventory Score (%)	29.62 (19.65)	30.47 (17.97)	28.00 (25.70)
Total Number of Symptoms (out of 22)	7.13 (7.06)	9.57 (7.38)	4.70 (5.88)
Time from Injury to Examination (days)	10.40 (27.16)	17.10 (37.43)	4.07 (6.43)

### Main results

Univariate analyses identified statistically significant relationships between headache (OR = 3.53; 95%CI = 1.08, 11.47), dizziness (OR = 2.14; 95%CI = 1.13, 4.02), cognitive impairments (OR = 2.72; 95%CI = 1.40, 5.28), clinical examination-cognitive testing (OR = 3.52; 95%CI = 1.08, 11.48), clinical examination-visual testing (OR = 2.98; 95%CI = 1.31, 6.80), and clinical examination-vestibular testing (OR = 4.28; 95%CI = 2.18, 8.43) and delayed return to activity. In all conditions, presence of these symptoms or test findings at initial clinical testing was associated with delayed clearance to activity (Table 3).

**Table 3:** Relationship between self-reported clinical symptoms and clinical examination findings and likelihood of delayed outcome^a^

Variable (symptom)	Odd Ratio (95% confidence Interval)	***P*** value
Headache	3.53 (1.08, 11.47)	**0.04**
Dizziness	2.14 (1.13, 4.02)	**0.02**
Neck Pain	0.99 (0.47, 2.01)	0.93
Cognitive impairments	2.72 (1.40, 5.28)	**< 0.01**
Photophobia	1.10 (0.54, 2.21)	0.79
Phonophobia	1.38 (0.64, 2.98)	0.40
Vision	0.98 (0.45, 2.13)	0.96
Clinical Examination - Cognitive testing	3.52 (1.08, 11.48)	**0.04**
Clinical Examination - Visual testing	2.98 (1.31, 6.80)	**< 0.01**
Clinical Examination - Balance testing	2.04 (0.86, 4.81)	0.10
Clinical Examination - Vestibular testing	4.28 (2.18, 8.43)	**< 0.01**
Clinical Examination - Cervical testing	2.03 (0.95, 4.34)	0.23

^a^Control for history of concussion, LD/ADHD, and Migraines (*N* = 163)

Multivariate hierarchical modeling identified the statistically significant relationships between cognitive symptoms (OR = 2.78; 95%CI = 1.29, 5.97) and clinical examination-vestibular testing (OR = 3.19; 95%CI = 1.49, 6.82) and delayed clearance to activity. In both cases, presence of these findings at initial evaluation was associated with delayed clearance to activity (Table 4).

**Table 4:** Multivariate analysis of relationship between self-reported clinical symptoms, clinical examination findings, and delayed outcome likelihood^a^

Variable (symptom)	Odd Ratio (95% confidence Interval)	P value
Headache Symptoms	1.60 (0.43, 5.99)	0.48
Dizziness Symptoms	1.87 (0.87, 4.04)	0.11
Cognitive Symptoms	2.78 (1.29, 5.97)	**0.01**
Clinical Examination - Cognitive	1.68 (0.43, 6.53)	0.45
Clinical Examination - Vision	1.77 (0.70, 4.47)	0.23
Clinical Examination - Vestibular	3.19 (1.49, 6.82)	**< 0.01**

^a^Control for history of concussion, LD/ADHD, and Migraines (*N* = 163)

### Secondary analyses

Sub-analyses of individuals with fewer days from injury to examination yielded three statistically significant findings in the multivariate modeling. A significant relationship was found between cognitive symptoms (OR = 4.90; 95%CI = 1.44, 16.64), clinical examination-vision (OR = 4.54; 95%CI = 1.18, 17.71) and clinical examination-vestibular testing (OR = 4.49; 95%CI = 1.34, 15.03) and delayed clearance to activity (Table 5). There were no significant relationships in the sub-analysis of individuals with longer days from injury to examination (Table 6).

**Table 5:** Multivariate analysis of sub-sample of patients who had fewer days from injury to examination^a^

Variable (symptom)	Odd Ratio (95% confidence Interval)	P value
Headache Symptoms	1.35 (0.12, 14.38)	0.80
Dizziness Symptoms	1.74 (0.54, 5.62)	0.35
Cognitive Symptoms	4.90 (1.44, 16.64)	**0.01**
Clinical Examination - Cognitive	1.26 (0.19, 8.23)	0.81
Clinical Examination - Vision	4.54 (1.18, 17.51)	**0.03**
Clinical Examination - Vestibular	4.49 (1.34, 15.03)	**0.02**

^a^Analyzing the relationship between report of clinical symptoms and clinical examination findings and the likelihood of a delayed outcome (*N* = 86)

**Table 6:** Multivariate analysis of sub-sample of patients who had extended (longer) days from injury to examination^a^

Variable (symptom)	Odd Ratio (95% confidence Interval)	P value
Headache Symptoms	0.74 (0.09, 5.58)	0.77
Dizziness Symptoms	2.41 (0.73, 7.93)	0.15
Cognitive Symptoms	1.86 (0.61, 5.84)	0.27
Clinical Examination - Cognitive	3.40 (0.31, 36.96)	0.32
Clinical Examination - Vision	0.65 (0.16, 2.63)	0.54
Clinical Examination - Vestibular	3.11 (0.91, 10.68)	0.07

^a^Analyzing the relationship between report of clinical symptoms and clinical examination findings and the likelihood of a delayed outcome (*N* = 77)

## Discussion

The objective of this study was to examine whether clinical examination results were associated with clearance to return to activity in individuals with concussion symptoms who were seen acutely at a concussion clinic. A secondary aim was to explore the role of early examination and how this influences the prognosis of the individual. Of the 163 individuals with concussion, 154 (94.4%) were 18 and under. Our data support that the use of selected clinical examination findings to capture specific clinical symptoms can identify the likelihood of a delayed recovery. Further, the strength of these associations appears to be mediated by fewer days between injury and clinical examination.

Symptoms of headache, dizziness, and self-reported cognitive impairments were associated with delayed recovery in the bivariate analysis, whereas only cognitive symptoms were statistically significant in the multivariate findings. Multivariate analysis of sub-sample of patients who had fewer days from injury to examination also found cognitive symptoms to predict delayed recovery. Others have identified the role of cognitive symptoms on delayed recovery [8, 31]. Brown conducted a prospective cohort study of patients who presented to a Sports Concussion Clinic within 3 weeks of injury and found from the variables assessed that total symptom burden at initial visit and cognitive activity level were independently associated with duration of symptoms [32]. Lau et al. reported that presence of on-field dizziness was associated with an odds ratio of 6.34 of protracted recovery after concussion [5]. This study associated acute dizziness with a 2.11 odds ratio of protracted recovery, but also found that headache and cognitive impairments had increased likelihood of delayed clearance with odds ratios of 3.48 and 3.65 respectively.

The clinical examination findings during cognitive testing, visual testing, and vestibular testing were associated with delayed recovery in the bivariate analysis, whereas only vestibular symptoms were statistically significant in the multivariate findings. Visual and vestibular examination findings were both statistically significant in the sub-analyses involving early examination after injury. Few others have studied the relationship between visual symptoms and delayed recovery. Heitger et al. compared 36 patients at 3–5 months with post concussive syndrome following a mild closed head injury to a cohort with normal recovery and found the post concussive syndrome group performed worse on anti-saccades, self-paced saccades, memory-guided sequences and smooth pursuit [33]. Maruta et al. looked at the presence of cognitive deficits in adults with persistent symptoms after a concussion and found they demonstrated increased gaze position error variability on visual tracking following an attention demanding task [34]. There is also paucity in the literature looking at the relationship between vestibular symptoms as predictors of delayed recovery follow concussion [35]. Zhou and Brodsky conducted a case series chart review of 42 pediatric patients with balance and/or vestibular complaints following sports-related concussions and found that 90% of the children with protracted dizziness or imbalance following sports-related concussion had at least one abnormal finding with the most frequent deficit found in dynamic visual acuity testing [36].

Current literature has looked at the role of timing in concussion management specific to removal from play and incidence of delayed recovery, but there are no reports of how time to clinical evaluation is associated with delayed recovery. Asken et al. conducted a cross-sectional study of 97 athletes who sustained a sport-related concussion and grouped them into immediate removal from activity or delayed removal from activity and found athletes in the latter group averaged 4.9 more days missed than those is the immediate removal group [37]. With respect to prognosis, the current findings support the importance of early examination after injury in mitigating a delayed recovery.

The current authors found that early examination had a mediating effect on the predictors and outcome variables. Interestingly, there were no predictors in the sample of patients who were involved in a later examination that were associated with delayed recovery. Past findings of studies that have failed to identify predictors may have been influenced by the sample of the patients and whether the individuals were seen earlier versus later following injury.

### Limitations

The study design as a retrospective analysis is one limitation. For this study, delayed recovery was associated with time to clearance. Delayed recovery was defined as individuals who took longer than the median value of time to clearance and not a clinically defined time point such as 14 days. To increase the understanding of the analysis, time to clearance was categorized by median values. Timing to clearance is a variable that is notably influenced by outside factors; factors that may not be related to the symptoms of the patient such as 1) physician variability, 2) access to care, 3) the nature of the activity that is needed for clearance, 4) psychosocial components related to return to school/sport. Further, the study used multiple providers which could influence the interpretation of clinical exams and consistency of performance of exams. Lastly, there may be consistency issues when reporting the presence of acute symptoms as the reliability of the report depends on the person completing the forms as well as the length of time from injury to evaluation.

## Conclusions

Selected symptoms and a standardized clinical examination provide value in identifying individuals who are likely to exhibit a delayed recovery. In particular, a positive finding during vestibular testing and presence of cognitive impairments identified at initial evaluation were associated with increased odds of a delayed clearance to return to activity. Our data also support the mediating influence of time to examination. Prognosis of delayed recovery may have greater utility when symptoms are captured and tests are performed early after onset of injury.

## Data Availability

The datasets analysed during the current study are not publicly available due to institutional restrictions regarding the accessibility of private health information. A limited dataset, with HIPAA identifiers removed, may be available from the corresponding author on reasonable request.
